# Normal Glucose Metabolism in Carnivores Overlaps with Diabetes Pathology in Non-Carnivores

**DOI:** 10.3389/fendo.2013.00188

**Published:** 2013-12-03

**Authors:** Thomas Schermerhorn

**Affiliations:** ^1^Department of Clinical Sciences, College of Veterinary Medicine, Kansas State University, Manhattan, KS, USA

**Keywords:** feline, dolphin, liver, pancreas, nutrition, carbohydrate, protein, animal models

## Abstract

Carnivores, such as the dolphin and the domestic cat, have numerous adaptations that befit consumption of diets with high protein and fat content, with little carbohydrate content. Consequently, nutrient metabolism in carnivorous species differs substantially from that of non-carnivores. Important metabolic pathways known to differ between carnivores and non-carnivores are implicated in the development of diabetes and insulin resistance in non-carnivores: (1) the hepatic glucokinase (GCK) pathway is absent in healthy carnivores yet GCK deficiency may result in diabetes in rodents and humans, (2) healthy dolphins and cats are prone to periods of fasting hyperglycemia and exhibit insulin resistance, both of which are risk factors for diabetes in non-carnivores. Similarly, carnivores develop naturally occurring diseases such as hemochromatosis, fatty liver, obesity, and diabetes that have strong parallels with the same disorders in humans. Understanding how evolution, environment, diet, and domestication may play a role with nutrient metabolism in the dolphin and cat may also be relevant to human diabetes.

## Introduction

Recently, it has been demonstrated that healthy Atlantic bottlenose dolphins (*Tursiops truncatus*) exhibit metabolic changes after fasting that are similar to those observed in humans with diabetes, including an elevation in fasting plasma glucose concentration (1, 2). The domestic cat (*Felis catus*), another obligate carnivore, exhibits metabolic characteristics during fasting that are similar to those observed in dolphins. A clinical syndrome of diabetes mellitus described in pet cats has been proposed as a model for human diabetes (3). In contrast with the dolphin and other marine mammals, glucose metabolism has been extensively studied in the cat under various conditions including clinically normal cats, obese cats, and cats with diabetes. Lessons learned from species such as the dolphin and the domestic cat may cast light on general metabolic pathways that have evolved to meet the unique glucose needs of carnivores as well as the defects in metabolism that contribute to diabetes development and progression in humans.

This article focuses on the similarities between the adaptive metabolism of carnivores and important features of diabetes pathology in humans. The objectives were to: (1) review adaptations in nutrient metabolism that have appeared during evolution in mammals that consume a meat-based diet that is naturally low in carbohydrate; (2) examine parallels between select metabolic features of normal carnivores and metabolic disturbances observed in humans with diabetes; (3) discuss the usefulness of carnivore species as diabetic models.

## Overview of Carnivore Carbohydrate Adaptations

### Definition of “carnivore”

For purposes of the following discussion, “carnivore” is used to describe any animal that consumes a diet composed of at least 70% animal tissue (4). While many familiar carnivores (e.g., obligate carnivores, such as felids, or scavengers, such as hyenas) are taxonomically grouped in the family Carnivora, some Carnivora species are distinctly omnivorous (some dog and bear species, for example) or even herbivorous (the giant panda) in their diet preferences. When defined by diet, phylogenetically diverse mammals such as dolphins and whales (family Cetacea) and insectivorous bats (family Chiroptera) are also carnivores.

### Feline adaptations to carnivory

The domestic cat is among the most extensively studied carnivores and serves as a representative model for metabolic adaptation to carnivory. Numerous anatomic, behavioral, and physiologic adaptations have appeared during feline evolution as a result of pressures imposed by the nutritional needs and feeding ecology of cats (5). Underlying the obvious anatomical features and behavioral traits that serve cats in their ecological role as apex predators are less obvious but equally important physiologic and metabolic adaptations. Feline adaptations to carnivory have been reviewed in detail by others (6–8) but a brief summary of the species’ unique adaptations related to carbohydrate metabolism is presented in the next section.

Cats do not exhibit a preference for sugars in the diet and at least one gene needed to detect the “sweet” taste is not expressed (9–11). The molecular basis for taste loss is disruption and pseudogenization of the *tas1R2* gene, which encodes one subunit of a heterodimeric sweet receptor (11, 12). Analysis of sweet taste preferences and *tas1r* gene isoforms from six carnivores (one felid and five non-felids) showed that only the felid (Asiatic lion) showed no taste preferences when offered water or water with added sweetener (13). The lion *tas1R2* gene, like the same gene in the cat, is a pseudogene (13). The same microdeletion in exon 3 of *tas1R2* is conserved in the domestic cat, Asiatic lion, cheetah, and tiger (12, 13). Additional taste loss is observed in pinnipeds (seals and sea lions), all species of which are carnivores. In eight pinniped species examined, the *tas1R3* gene was found to be a pseudogene (14). Lack of *tas1R3* prevents formation of the unami receptor, a heterodimer of *tas1R1* and *tas1R3* that detects the savory taste (unami) in protein-rich foods (15). The relationship between a carnivorous diet and the evolutionary loss of sweet taste is underscored by the observation that canids, which are close phylogenic relatives of seals but are not obligate carnivores, retain the sweet receptor.

Other features of feline metabolism that are relevant to carbohydrate metabolism have been determined from physiology studies. Cats are reported to lack salivary amylase, which is necessary for initiating digestion of some forms of carbohydrate (16). Although cats are able to digest and absorb carbohydrates, including simple sugars, the literature contains reports that show cats have reduced activities of pancreatic amylase and intestinal disaccharidases when compared to other species (9, 17–19). Interestingly, certain important features of feline intestinal carbohydrate digestion and absorption, such as carbohydrate transport and disaccharidase activity, appear non-adaptable in the face of fluctuations in dietary carbohydrate content (20). Despite the unique adaptations observed in cats, it is clear that cats can efficiently digest carbohydrates. Diets containing >40% digestible carbohydrate can be tolerated by cats. Digestibility of most sugars is >90%, including simple dietary carbohydrates, such as glucose and fructose (20, 21).

### Protein metabolism is linked to glucose metabolism

The dietary protein requirement for carnivores exceeds that of omnivores and herbivores (22). In “hypercarnivores,” such as the domestic cat and dolphin, the percentage of dietary protein is at least 70% when animals ingest a natural, prey-based diet (4). The metabolic adaptations of cats with respect to protein metabolism have been studied in greater detail than in other carnivores. Like most mammals, cats must derive essential amino acids from the diet but strict carnivory poses special problems. Specific and unique requirements for arginine and taurine in cats illustrate the dietary inflexibility imposed during evolution on strict carnivores. Unlike most mammals, cats are unable to synthesize arginine, a vital intermediate in the urea cycle, and as a result have an absolute requirement for dietary arginine. However, the cat is exquisitely sensitive to arginine deficiency. Without dietary arginine the urea cycle does not function and the accumulation of nitrogenous byproducts results in the rapid development of hyperammonemia (within hours), which may be fatal (23). Along with a high dietary protein requirement, cats also have comparatively greater nitrogen loss and hepatic activities of catabolic enzymes than non-carnivores (22). Sentinel studies by Rogers et al. established a paradigm for feline protein metabolism that was challenged only recently (24). The early work of Rogers et al. showed that the rate of protein metabolism did not vary with dietary protein content in cats (24). Based on recently published reports, it now is clear that activities of pyruvate carboxylase, fructose-1,6-bisphosphatase, and glucose-6-phosphatase (G6Pase) do modulate in response to dietary protein content but enzyme adaptation is incomplete when dietary protein is below required minimum content (about 15% metabolizable energy in cats) (25, 26).

Protein metabolism in carnivores is intimately linked to hepatic glucose metabolism because protein catabolism provides substrates that channel into gluconeogenic pathways. The strong interdependent relationship between these two pathways is postulated to have evolved as a means to provide glucose needed to maintain brain function in the face of low dietary availability of carbohydrates (8, 27). With respect to body size, the brain of felids is proportionately large and as a result, consumes a higher portion of glucose when compared to non-felids of similar size (8). Allometric scaling of the brain has occurred over the course of dolphin evolution, too. The dolphin has an encephalization quotient (a measure of the brain to body size that allows comparison between different species) that is surpassed only by humans and non-human primates (28). The continuous need for glucose to provide substrate for oxidation reactions that supply energy for neuronal function requires either a constant source of absorbable carbohydrate or sustained endogenous production of glucose via breakdown of stored carbohydrate (glycogenolysis) or *de novo* production from non-sugar substrates (gluconeogenesis). Lacking substantial dietary carbohydrate, cats use dietary protein to supply brain energy needs via the latter mechanisms (8).

The need for large amounts of glucose to fuel brain function not only requires a continuous source of glucose, but has led to adaptations that improve glucose delivery to the brain. There is evidence from dolphins and related toothed whales (odontocetes) for the presence of erythrocyte adaptations the support transport of large amounts of glucose in the blood (2). In most mammals, erythrocyte glucose concentrations are lower than the plasma concentration, but in odontocetes, humans, and non-human primates, rapid transmembrane glucose uptake by erythrocytes produces equivalent intracellular and plasma glucose concentrations (29). Glucose uptake in the erythrocytes of dolphins is GLUT 1-mediated as in humans (30). GLUT 1 is a widely expressed member of the GLUT family of mammalian sugar transport proteins. GLUT 1 is a high affinity transporter that widely expressed in cells, particularly those that depend on glucose for energy, such as the brain and RBCs (31). The high affinity for glucose means that GLUT 1 activity is saturated at basal blood glucose concentration (about 5 mM in mammals), so it is the amount of GLUT 1 expressed on the plasma membrane that is the principal determinant of a tissue’s transport capacity (31). It is not known whether the erythrocytes of all carnivores are similar to those of dolphins. As a marine mammal, the same erythrocyte adaptations that permit large capacity glucose transport may provide additional physiologic advantages, such as supplying fuel during prolonged dives, which are not required by terrestrial carnivores. Further, the *Tursiops* EQ is among the highest found in mammals outside of humans and is higher than many non-human primates (28, 32). The cat EQ, however, is about fourfold smaller than the dolphin (33). The very high EQ of the dolphin and related odontocetes may impose additional demands on glucose availability than those of the cat, despite the relatively large brain of felids.

Hepatic carbohydrate metabolism of cats has several characteristics that distinguish it from metabolism of non-carnivores but are not necessarily unique. A principal feature of feline hepatic glucose metabolism is the absence of glucokinase (GCK) activity and the predominance of low *K*_m_ hexokinase (HK) activity (34, 35). The enzyme pattern in the liver of the cat is not unique among mammals or even among carnivores. There are four mammalian hexokinases (identified as HK I–IV; HK IV is also known as GCK) that are expressed in various patterns in the liver but a particular pattern is not always consistent within taxonomic Orders [for summary, see Ref. (36)]. For example, the hexokinase pattern is variable between members of Carnivora, while the pattern is relatively consistent in Artiodactyla. In cat liver, hexokinase I and II are present but GCK is absent. The pattern in the cat is identical to a bat from a separate phylogenic order (Carnivora and Chiroptera, respectively) but differs from that found in the dog (HK I, HK II, and GCK), also in the order Carnivora. The spectrum of hexokinases present in the liver likely has functional consequences, particularly the presence or absence of GCK (HK IV). Studies of hexokinase distribution established that the lack of hepatic GCK is more consistent with the dietary preference of a particular species than with the evolutionary relationship between species. The relationship between GCK and diet preference is borne out in lower vertebrates, too. The barn owl and trout, both carnivores, also lack hepatic GCK (37, 38). It is of interest to note that induction of hepatic GCK activity after feeding a high carbohydrate diet has been shown to occur in trout and chicken, species previously reported to lack hepatic GCK (39, 40), but induction has not been studied in the cat.

Gluconeogenesis in the non-carnivore liver is activated during fasting and starvation and is inhibited by nutrient and hormone signals from the gut after a meal (40). In the cat, hepatic gluconeogenesis is reported to be continually active (24). Consistent with the need for a high gluconeogenic capacity, the activity of the G6Pase enzyme system is comparatively high in feline liver compared to canine liver as are activities of other rate limiting gluconeogenic enzymes, pyruvate carboxylase, and fructose-1,6-bisphosphatase (24, 34, 35). The mink (*Mustela vison*), an obligate carnivore, also exhibits active hepatic gluconeogenesis. In fact, activities of G6Pase and PEPCK are higher than measured in cat liver (41). Comparatively, gluconeogenic enzyme activity in both the cat and mink exceed activity in rat liver (41).

Rates of gluconeogenesis and protein catabolism in the feline liver do not adjust completely to changes in dietary composition (24–26). The constant requirement for substrate to supply high rates of protein oxidation and gluconeogenesis is hypothesized to underlie the high dietary protein requirement of cats. Several lines of evidence now indicate that feline hepatic metabolism is capable of being regulated by dietary components. An *in vitro* study with isolated hepatocytes obtained from cats fed diets of different protein composition showed a parallel increase in protein oxidation rates and protein intake, although the gluconeogenic rates did not vary with protein intake (42). Tracer and indirect calorimetry studies, respectively, revealed enhanced glycogenesis in liver of cats in response to a rise in the insulin level (43) and increased glycogen and lipid production along with enhanced glycolysis in lean cats under euglycemic clamp (44). Recent work using *in vivo* nuclear magnetic resonance (NMR) to study adaption to obesity and diet yielded unexpected results. Despite insulin resistance and impaired glucose tolerance, chronically obese cats exhibit lower hepatic glucose production during fasting than lean counterparts (45, 46).

## Carnivore Hepatic Glucose-Sensing Pathways

The hepatic glucose-sensing pathway, which is important for normal glucose homeostasis and disordered in diabetes, has been studied at the molecular level in the domestic cat. The hepatic glucose-sensing pathway illustrates how normal carnivore metabolism may inform understanding of the abnormal glucose metabolism that characterizes the diabetic state.

### The concept of glucose sensing

In mammals, blood glucose concentration is maintained within narrow physiologic limits by balancing glucose entry into the circulation with its removal by the tissues. Regulatory mechanisms are needed to ensure an adequate supply of glucose to tissues, such as the brain, that require high amounts of glucose for normal function and to guard against detrimental fluctuations in glucose concentrations. The endocrine pancreas (islets of Langerhans) and liver are major organs involved in blood glucose sensing, but important roles are hypothesized for specialized neurons in the brain and enteroendocrine cells in the gut. Precise minute-to-minute regulation of insulin secretion from the pancreatic beta cells requires a glucose-sensing mechanism to modulate beta cell function according to the prevailing glucose concentration, while glucose-sensing pathways in hepatocytes ensure proper balance between hepatic glucose usage and production (47). Central to both glucose-sensing mechanisms is the concept of a molecular “glucose sensor” as the principal regulator of cellular glucose flux and, thus, the generation of downstream signals that adjust cellular function (47).

An important molecular pathway involved in mammalian glucose-sensing involves the enzyme GCK. Mammalian GCK, the protein product of the highly conserved *GCK* gene, is found only in glucose-responsive tissues where it is the *de facto* “glucose sensor” (48). *GCK* is present as a single copy in mammalian genomes under the control of two separate promoters (49). The “neuroendocrine” (or “pancreatic”) promoter controls expression in pancreatic islet cells and specialized cells in the gut and hypothalamus (48, 49). The second promoter, the “hepatic” promoter, is active only in hepatocytes (48, 49). The major *GCK* mRNA isoforms present in pancreas and liver differ at the 5′-UTR and exon 1; the remainder of the mRNAs containing exons 2–10 and the 3′ regions are identical (48). Minor mRNA isoforms with alternate first exons have been found in human and rat liver (50, 51), suggesting the pattern of hepatic *GCK* expression may vary in some species. Differential *GCK* expression by hepatocytes and pancreatic endocrine cells conveys the ability to vary the metabolic response according to the blood glucose concentration (49). The importance of *GCK* expression for maintenance of glucose homeostasis and as a crucial component of glucose-sensing and metabolic regulation has been firmly established in experimental and clinical over the past several decades, including the study of naturally occurring *GCK* mutations of humans (52).

### Glucose sensing in the pancreas

A complete review of the cellular pathways involved in the beta cell response is beyond the scope of this article but these pathways have been well established in human and rodent beta cells and in insulin-secreting cell lines (53–56). *GCK* as an important and essential component of beta cell glucose sensing is supported by a large body of evidence from overexpression studies (57), site-directed mutagenesis (58, 59), and study of naturally occurring mutations (60, 61), knockout models (62–64), and pharmacologic manipulation of GK function (65, 66).

Although detailed studies are lacking, the pancreatic glucose-sensing mechanism is likely to be fundamentally similar in carnivores and non-carnivores. *GCK* mRNA has been detected in the feline pancreas (7, 35, 66) and in an insulin-producing pancreatic tumor from a cat (7). GCK activity in normal feline pancreas has yet to be confirmed. However, in a cat with a functional insulin-producing tumor and hypoglycemia, tumor *GCK* expression was nearly 17 times greater than normal pancreatic tissue from the same cat, suggesting a relationship between the magnitude of *GCK* expression and insulin secretion, similar to that observed in *in vitro* overexpression studies (7). Feline islets also express numerous other genes with roles in insulin signaling (67). Furthermore, pharmacologic treatment with arginine (68, 69), sulfonylureas (70), or exenatide (a GLP-1 mimetic) (71) also elicit the expected increase in insulin secretion in cats. At present, there is no evidence that beta cell glucose-sensing or stimulus secretion coupling differ between carnivores and non-carnivores. When viewed in light of results from *in vivo* clinical and experimental studies of glucose-stimulated insulin secretion in cats, the importance of glucose as a major physiological stimulator of insulin secretion in cats is affirmed.

### Overview of hepatic glucose sensing

In non-carnivore models, including rodents (72) and dog (73, 74), a network of interacting hepatocellular proteins serves to sense changes in glucose and other nutrients after a meal and initiates the appropriate metabolic response. The “fasted-to-fed” metabolic transition in the liver is mediated by nutrients, especially carbohydrates, and hormones, especially insulin and glucagon (40). In the fasted state, when blood glucose and insulin concentrations are basal, the insulin:glucagon ratio favors hepatic glucose production via gluconeogenesis. After ingestion of a carbohydrate-containing meal and stimulation of pancreatic insulin secretion, the combination of elevated portal glucose concentration and the increased insulin:glucagon ratio promotes hepatic glucose uptake via stimulation of GCK activity and simultaneously reduces hepatic gluconeogenesis via inhibition of G6Pase activity. Circulating hormones and nutrients produce their effects on hepatic function by directly altering protein interactions and enzymatic activity but exert equally important long term effects on hepatic glucose metabolism by influencing the expression of genes involved in the glucose-sensing network.

#### Molecular glucose sensing in the non-carnivore liver

Hepatic GCK activity is influenced by nutritional status and acute changes are observed soon after feeding. Dietary nutrients, especially glucose and fructose, acutely stimulate hepatic GCK activity. Within individual hepatocytes, GCK activity is also regulated by the glucokinase regulatory protein (GKRP), which is localized in the hepatocyte nucleus. GCK undergoes a well-described nuclear-cytosolic translocation cycle and its subcellular localization varies with the nutritional state (75). In the fasted state, GCK is bound to GKRP and localized within the hepatocyte nucleus, where it is functionally inactive (76). After feeding, GCK translocates to the cytosol and becomes enzymatically active (76). GCK entry into the nucleus requires GKRP but exit from the nucleus is mediated by a nuclear export signal in the GCK amino acid sequence (77). The GCK/GKRP regulatory unit provides a mechanism for hepatocyte metabolism to respond to demands for increased glucose phosphorylation. There are strong reciprocal control effects of glucose phosphorylation (GCK exerts a positive effect; GKRP exerts a negative effect) and *GCKR* overexpression inhibits glucose phosphorylation and decreases GCK activity (78).

#### Molecular glucose sensing in the carnivore (feline) liver

Although feline genes involved in glucose metabolism appear to be highly conserved (79–81), work in cats has clearly demonstrated that the molecular basis of feline hepatic glucose-sensing differs from the model defined in the non-carnivore liver (82). *GCK* and *GCKR* (the gene that encodes GKRP) mRNAs are absent from feline liver and activities of GCK and GKRP proteins are not detectable (35, 38, 82). The cat appears to be a naturally occurring model for *GCKR* knockout. The phenotype of transgenic *GCKR* knockout mice is normal (normal blood glucose and no evidence of diabetes). However, like the cat, *GCKR* knockout mice are susceptible to stress-induced hyperglycemia and exhibit mild glucose intolerance compared with wild-type that express *GCKR* (83). Heterogeneous or homogeneous *GCKR* knockouts show reduced liver GCK activity (83).

Lack of GCK and GKRP suggests that feline liver cannot respond incrementally to physiologic changes in blood glucose. In the absence of GCK, the initial glucose phosphorylation step must be performed by another hexokinase (e.g., HK I). The kinetic features (high affinity, low *K*_m_) of hexokinase I means that enzyme activity will be maximal at glucose concentrations below the basal blood glucose concentration and cannot increase its rate of glucose phosphorylation in the face of increasing blood glucose levels (e.g., after a meal). However, GKRP has effects on *GCK* gene and protein levels distinct from its action as a regulator of GCK activity. Thus, the lack of *GCKR* expression in cats may contribute to reduced *GCK* mRNA expression and low enzyme activity observed in feline liver as was shown for mice with *GCKR* knockout (84).

Hepatic expression and activity of fructokinase (*KHK* gene), which metabolizes fructose and serves as an upstream regulator of GK:GKRP binding, is preserved in the cat. Although fructose can be digested and absorbed by cat intestine (20), it is somewhat unexpected to find that fructokinase activity in feline liver is comparatively higher than in canine liver (35). The reason for abundant fructokinase expression in the feline liver is not clear since fructose, a simple sugar, is not expected in substantial quantity in a carnivore diet. KHK is widely expressed in feline tissues, including tissues (e.g., spleen) that have no role in nutrient metabolism (80). A similar pattern of KHK expression was previously defined in non-carnivore mammals (85), which led investigators to hypothesize possible roles for KHK in cell function in addition to its role in dietary fructose metabolism (85, 86).

The importance of *GCK* expression for normal glucose metabolism has been established beyond doubt by transgenic and gene knockout studies and the study of naturally occurring *GCK* mutations in humans [reviewed by Osbak et al. (52)]. Transgenic mice and *in vitro* studies show that even small reductions in the level of GCK expression can have dramatic negative impact on glucose sensitivity while *GCK* overexpression generally enhances glucose sensitivity (87). Transgenic mice with global *GCK* loss or targeted disruption of islet *GCK* expression are diabetic at birth and die within a few days (87). Mice with targeted disruption of hepatic *GCK* are viable and do not exhibit a diabetic phenotype but have altered glucose tolerance and display hyperglycemia under stress conditions (87).

Over 600 naturally occurring mutations in *GCK* have been described (52). Activating mutations are associated with hyperinsulinemia and hypoglycemia, which lead to a group of syndromes commonly referred to as congenital hyperinsulinemias (CHI). Inactivating mutations result in impaired insulin release and hyperglycemia and are associated with familial forms of diabetes. *GCK* mutations cause a monogenetic form of human non-insulin dependent diabetes known as Maturity Onset Diabetes in the Young (MODY). Each MODY subtype (there are five subtypes) results from mutation of a gene involved in glucose metabolism; *GCK* mutations cause MODY type 2 (MODY2), which account for as many as 60% of all MODY cases (7). Along with its causative role in MODY-GCK, a monogenic form of diabetes, *GCK* has long been suspected as a candidate gene with a role in the development of other forms of diabetes mellitus, too. Although several investigations have linked *GCK* mutations and polymorphisms to human type 2 diabetes, at present a clear role for GCK in the development of human type 2 diabetes has not been demonstrated (88–90). No disturbances of glucose metabolism due to GCK mutation have been reported in dolphins, cats, or any other wild or domestic mammals, however, newer technology for rapid, inexpensive sequencing of individual genomes may render discovery of such mutations more likely in the future. Identification of genetic polymorphisms and study of naturally occurring mutations can provide valuable insight into the functional role of genes in glucose metabolism pathways of carnivores.

Although reduced capacity for metabolism of simple sugars is consistent with consumption of a low carbohydrate diet, in light of the importance of *GCK* expression in humans and rodents, the complete lack of hepatic GCK activity remains difficult to reconcile from a physiological perspective. For example, *GCK* expression has been demonstrated to be crucial for coordination and regulation of hepatic glycolysis and lipogenesis in mouse hepatocytes (91). In the absence of GCK, glucose phosphorylation in hepatocytes is catalyzed by HK I, a hexokinase with kinetic features that render it unsuitable as a replacement for GCK. GCK shows an activity curve that increases with the glucose concentration and activity is nearly linear over the physiologic range of glucose (92). HK I activity is maximal at basal glucose concentration and the enzyme is unresponsive to changes over the physiologic range (92). Further, unlike GCK, HK I is inhibited in the presence of its reaction product, G-6-P, which is continually produced by gluconeogenesis in feline hepatocytes (92). Thus, the most frequently proffered explanation that lack of GCK expression is overcome by upregulation of HK activity is unlikely from a biochemical perspective and lacks mechanistic detail. A clear challenge for future research seeking to understand carnivore carbohydrate metabolism is determination of the mechanism(s) by which hepatic glucose phosphorylation can proceed efficiently in the absence of GCK. Until carnivore studies become available, results from transgenic studies that aimed to ameliorate diabetes in rodent models suggest possible mechanisms.

Activation of glucose disposal pathways that are distal to the GK-mediated phosphorylation step promotes hepatic glycolysis and glycogen synthesis; some of these improvements occur even when GK activity is nearly absent (40). Overexpression of Protein Targeting to Glycogen (PTG) in 3T3 LI cells or rat hepatocytes increases glycogenesis (93, 94). Interestingly, glycogen synthesis in rat hepatocytes overexpressing PTG is glucose and insulin independent and continues even when glucose is excluded from culture media (94). Lacking glucose, carbon incorporated into glycogen is likely contributed by gluconeogenic pathways (94). The results from expression studies suggests that PTG-mediated activation of glycogenesis is one possible mechanism by which feline hepatocytes could synthesize glycogen when the dietary glucose concentration is low, yet there are high concentrations of gluconeogenic precursors.

The action of glycogen targeting proteins may inform another important but unanswered question regarding feline hepatic carbohydrate metabolism. Several studies have concluded that the improved glucose utilization and glycemia observed in diabetic models when PTGs are overexpressed is due to a “pull” effect that redirects G-6-P away from gluconeogenic pathways and into glycogen synthesis (40). The same improvements are observed in the SMZ-induced diabetic rat model in which GK activity is very low (95). In the latter model, it may be that the enhanced flux of G-6-P through glycogenic pathways is enough to disinhibit hexokinase I and permit an increase in the glucose phosphorylation rate (95). Comparative overexpression of PTGs is a possible mechanism to explain how the feline liver could continue to funnel glucose through the G-6-P pathway despite the lack of *GCK* expression. Despite the low carbohydrate content of the natural feline diet, glycogenic pathways are fully functional in feline hepatocyte. A recent report stating that the glycogen storage capacity in feline liver is comparable to that of non-carnivores (dog and human) (96) lends support to the possibility that PTGs may make enough G-6-P available to support robust glycogenesis. While it is intriguing to contemplate the involvement of PTG-mediated pathways in the feline glucose metabolism, the expression of PTG and related proteins in carnivore hepatocytes and their role(s) in modulating glycogenesis remains an unexplored area that awaits investigation.

### Is the cat a representative model for carnivore glucose sensing?

The lack of *GCK* and *GCKR* expression in liver of the domestic cat has been convincingly demonstrated. But is the lack of expression of these genes unique to the cat or is it a universal feature of carnivore metabolism? Direct determination of *GCKR* expression or activity in vertebrate liver has not been widely explored. A study by Vandercammen and van Schaftingen (38) showed that species (cat, trout, cow, goat, chicken) that lacked GCK activity also lacked GKRP activity. Recently, Wang el al. (97) used bioinformatics to examine the *GCKR* gene in the genomes of carnivore and non-carnivore species. Using published gene sequences and available genomic assemblies to predict gene sequences, it was found that *GCKR* was intact in most non-carnivore species. However a variety of *GCKR* mutations were found in the genomes of mammalian carnivores, including the cat and dolphin (97). Feline *GCKR* was predicted to have frame shift mutations in three separate exons (exons 2, 4, and 10) and a splice mutation in exon 2. *GCKR* in *Tursiops* had predicted frame shift mutations in seven exons (exons 1, 3, 4, 6, 9, 15, and 19) and a splice mutation in exon 1 (97). At least one mutation that is likely to disrupt *GCKR* coding is present in species that have confirmed absence of hepatic GCK expression or activity. However, no single mutation is shared by species with inactivated *GCKR*. A recent survey of *GCK* gene structure in all carnivore and non-carnivore species for which genome sequences are available concluded that *GCK* is a highly conserved (81). It was predicted that *GCK* is a functional gene (if expressed) in all of the mammals examined (81). The finding that *GCK* is conserved and predicted to be functional in carnivores, led Wang et al. to hypothesize that it was the presence of mutations that disrupt *GCKR* expression that lead to a parallel loss of *GCK* expression in carnivores (97). The genetic mechanism(s) linking the two genes is unknown but is likely to be complex. The consistent loss of *GCKR* and *GCK* in carnivore species suggests the change is adaptive. The observation that different mutations produce a common phenotype in different carnivores suggests *GCKR* mutations arose separately during evolution. In the cat and dolphin (and likely other carnivores), separate genetic events leading to the evolutionary loss of *GCKR* likely represents independent adaptations to carnivory. Like *GCKR*, other hepatic genes with metabolic roles appear to have adapted to serve carnivore metabolism during evolution of carnivory (98, 99). Despite the apparent convergence of molecular mechanisms for carbohydrate metabolism observed in carnivores of different phylogenic origins, the possibility that individual carnivore species have evolved unique mechanisms in response to metabolic challenges should not be dismissed and emphasizes the continued need for species-specific investigations.

## “Diabetes Pathology” in Normal Carnivores

Normal glucose and protein metabolism of carnivores affects serum glucose and insulin in ways that resemble diabetes pathology in humans. Of course, nutrient metabolism observed in healthy carnivores, such as dolphins and cats, is “abnormal” or unusual only when compared with the same metabolic processes in non-carnivores. When viewed from a non-comparative perspective, carbohydrate and protein metabolism of healthy carnivores reflects processes which have appeared during evolution as adaptations to the consumption of diets naturally low in carbohydrate. In addition, some domesticated and captive carnivores develop pathologic conditions with similarities to human diabetes. Results of carnivore studies highlight the potential of these animals to be natural models for the study of common pathologies associated with human diabetes (1, 3, 100).

### “Glucose intolerance” in healthy cats and dolphins

It is reported that the bottlenose dolphin, *T. truncatus*, demonstrates comparatively high fasting blood glucose levels and exhibits prolonged glucose clearance compared with the same measures in humans (1, 2). These features of dolphin carbohydrate metabolism have parallels in the healthy cat, another obligate carnivore that exhibits mild carbohydrate intolerance after a glucose challenge and is prone to develop physiologic hyperglycemia (101, 102).

Venn-Watson and Ridgway (2) carried out hematologic and biochemical analysis of a large number of blood samples (*n* ∼ 1100 samples) obtained from Atlantic bottlenose dolphins that revealed significant differences between non-fasted (meal consumed within previous 6 h) or fasted (meal withheld for 10–14 h) conditions. In fasted dolphins, a slight but significant increase in glucose was detected. Fasted samples also had elevated platelet count, chloride and creatinine concentrations, and increased activities of serum alkaline phosphatase, gamma-glutamyl transferase, and creatine kinase. Fasting decreased serum concentrations of blood urea nitrogen, potassium, carbon dioxide, triglycerides, and uric acid. Changes in glucose, uric acid, GGT, ALP, platelet count, and acid-base indicators observed in fasted samples paralleled changes observed in humans with diabetes. A follow-up study by the same investigators examined dynamic glucose responses in dolphins fed either a dextrose solution or a fish meal (1). Both meals elevated serum glucose for 5 h post-ingestion but glucose remained elevated over baseline for up to 10 h in dolphins that ingested the dextrose solution. Along with delayed return to baseline, the peak mean glucose concentration at 5–10 h following dextrose ingestion was nearly 40% greater than that observed after the fish-based meal (209 versus 124 mg/dL, respectively) (1). Insulin and glucagon responses to a protein meal were determined in five male and female dolphins ranging in age from 8 to 48 years. Mean basal insulin in this group was ∼12 μIU/ml but mean insulin 2 h-post-prandial was lower (∼9 μIU/ml) (1). These data are difficult to interpret because insulin levels in this small group showed substantial variability, particularly the post-prandial measurements, possibly due to influences of gender and age, as noted for other hematologic and biochemical parameters (103). Further study with larger groups and uniform populations is needed to place these initial findings into context.

Oral glucose challenge tests with healthy dolphins yielded results that strongly suggest glucose intolerance when compared to similar challenges with non-carnivore species (1). First, mean blood glucose after a dextrose challenge took as long as 10 h to return to baseline in healthy dolphins. The time course to return to baseline after glucose challenge in the dolphin is much longer than expected in a human, rodent, or dog. Second, examination of blood glucose curves from individual dolphins given dextrose showed a rapid rise in glucose that reached peak concentration 4–8 h after ingestion. In contrast, glucose decreased hourly for 5–10 h after ingestion of a mackerel meal. Lastly, peak glucose concentrations achieved after dextrose ingestion in the dolphin are compatible with mild diabetes and were accompanied by an eightfold increase in urine glucose concentration, another feature of overt diabetes in humans.

Insulin and glucose responses have also been studied in cats fed diets containing various amounts of carbohydrate. Because commercial feline diets, even those with abundant carbohydrate content, contain very low amounts of simple sugars, such as glucose and fructose (104), glucose concentrations may rise slowly after a meal as complex carbohydrates undergoes the digestive process (105). As a result the post-prandial glycemic response remains poorly defined in cats with some studies reporting a blunted post-prandial hyperglycemia (21, 106). Intravenous infusion of large amounts of glucose (up to 1 g/kg body weight) induces a rapid rise in blood glucose and invokes insulin secretion. Intravenous glucose tolerance tests have typically shown delayed glucose clearance in normal cats (about 90 min) compared with dogs (about 40–60 min) and humans (30–40 min) (105, 107). The IVGTT produces a rapid glucose change that would not occur if the cat were consuming its natural diet. In bypassing the oral route, the IVGTT response does not account for potential contributions from gut hormones, such as GLP-1, to enhance the insulin secretory response. An interesting study by Hewson-Hughes et al. attempted to sidestep the inherent problems associated with previous approaches by comparing the glycemic response of a high protein diet with the same diet that had been “glucose-loaded” by the addition of d-glucose (105). Cats that ate the high protein diet + glucose showed markedly different post-prandial glucose and insulin patterns than did control cats that ate the unaltered diet. Cats consuming the glucose-loaded diet experienced large and sustained increases in glucose, with a mean peak concentration of ∼180 mg/dL (5 mM) and a 5- to 6-h duration of hyperglycemia. Curiously, despite marked hyperglycemia it produced, the glucose-loaded diet elicited a weak insulin secretory response, although insulin concentration still exceeded that recorded in cats eating the high protein diet without added glucose (105). Hyperglycemia in the diet + glucose group could develop as a result of insulin resistance, impaired hepatic glucose uptake, unsuppressed hepatic glucose output, or a combination of factors. The weak insulin secretory response to dietary glucose was unexpected and is unexplained considering that adequate insulin responses have been observed in cats consuming diets of varying carbohydrate compositions. It is unlikely that diminished pancreatic glucose-sensing accounts for the sluggish insulin response in the cat, but the finding deserves closer study.

In summary, post-prandial serum glucose and insulin fluctuations observed in healthy dolphins and cats would be consistent with mild degree of glucose intolerance if the same changes were found in non-carnivore species. Caution is warranted when comparing glucose tolerance between healthy individuals of different species (e.g., human versus dolphin). Such comparisons are useful and can inform understanding of normal carbohydrate metabolism for a particular species but differences should not be interpreted to imply pathology in one or the other species. The value in gaining and understanding of the mechanisms that govern glucose and insulin responses in healthy carnivores is the insight provided into the possible pathologic mechanisms that produce similar changes leading to glucose intolerance in non-carnivore species.

### Unsuppressed hepatic glucose output

In the diabetic liver, failure of insulin to induce a switch from glucose production to glucose utilization reflects hepatic insulin resistance (108–110). The blunted response to the rise in the insulin:glucagon ratio that occurs following a meal means that the diabetic liver continues to produce glucose in the post-prandial period when intestinal absorption of carbohydrate is also increased. These factors produce post-prandial hyperglycemia which is manifest clinically as glucose intolerance.

In carnivores consuming a natural diet, the low carbohydrate content minimizes the possibility that large changes in glucose will occur during the absorptive phase following a meal. When the diet contains low amounts of glucose, hepatic gluconeogenesis is predicted to be the major pathway to maintain blood glucose. Consistent with the latter expectation, the activities of key gluconeogenic enzymes (G6Pase, fructose-1,6-bisphosphatase, and pyruvate carboxylase) are increased in the liver of normal cats (34, 35). Whole animal studies in cats have shown that endogenous glucose production during fasting is mainly from gluconeogenesis but glycogenolysis contributes ∼30% of total glucose produced under fast conditions and as much as 40% in the post-prandial period (45, 96). The finding that “stress hyperglycemia,” a clinical phenomenon observed in pet cats presenting to veterinarians for treatment, is caused by enhanced hepatic glucose output, rather than insulin resistance as previously postulated, underscores the gluconeogenic potential of the feline liver (101). There is abundant evidence for robust glucose production in healthy cats during fasting and it is possible that physiologic inhibition following food intake is incomplete, although hepatic glucose metabolism is now recognized to be moderated to a greater degree than previously believed (45).

## Pathologic Disorders of Carnivores Associated with Insulin Resistance

Insulin resistance occupies a prominent pathophysiologic role in the development and propagation of the diabetic state. Insulin resistance can be conceptually defined as a resistance (impaired sensitivity) to insulin action at the tissue level. Insulin resistance is mediated at the cellular level with muscle being the most important affected tissue, but adipose tissue and liver also affected. Early insulin resistance is characterized by hyperinsulinemia but not hyperglycemia. Hyperglycemia does not develop because the reduced effect of insulin at target tissues is offset by an increase in insulin secretion. Later, when beta cell function is impaired and the total insulin secretory capacity falls, hyperglycemia develops because insulin resistance is now accompanied by a relative or absolute insulin deficiency.

In animal studies, insulin resistance is difficult to measure directly except under carefully controlled conditions using euglycemic clamp methods and is not routinely performed outside of the research setting. The euglycemic clamp is the gold standard used to measure insulin resistance in humans, too, but algorithms have been developed that use simple clinical measures to identify insulin resistant humans (111). The algorithms used in humans were generated from a large amount of data from human clamp studies and are not likely to be useful in other species. Lacking similar species-specific algorithms, insulin resistance in the cat or dolphin must be either measured using clamp methodology (112, 113) or inferred when glucose intolerance or reduced insulin responsiveness are detected (114, 115).

### Hemochromatosis and fatty liver in dolphins

Dolphins develop two hepatic disorders, hemochromatosis and fatty liver, that have been associated with acquired insulin resistant states in humans. Hemochromatosis, also known as iron overload, is a disorder associated with hepatic iron accumulation and severe insulin resistance (116). Hemochromatosis occurs in humans and has been documented in a variety of animals (117–119). Iron overload increases serum iron and ferritin concentration; ferritin increases in parallel with serum iron and can be used as an indicator for serum iron concentration. Affected dolphins have elevations in the serum iron and ferritin levels (120) and exhibit elevation in serum insulin and glucagon concentrations 2-h after feeding, consistent with insulin resistance (1). Iron overload may have a genetic basis, which is the most common etiology of human hemochromatosis, or may be an acquired disorder associated with a variety of non-hereditary conditions (121). Phlebotomy is the treatment of choice for hemochromatosis in dolphins and the intervention successfully decreases serum iron levels (122). Interestingly, insulin resistance – as defined by detection of persistent post-prandial hyperinsulinemia – persists in treated dolphins despite normalization of serum iron and ferritin levels (121, 122).

Fatty liver of dolphins resembles human non-alcoholic fatty liver syndrome and hepatic lipidosis described in cats and other veterinary species. A recent investigation that examined archived pathology samples from 18 captive dolphins that had died over a 25-year period reported finding histological evidence of fatty liver in nearly 40% of dolphins (116). Lipidosis was found in association with other liver disorders (hemochromatosis and hepatitis) in some dolphins (116). Hepatic accumulation of lipid occurs when lipid mobilization exceeds hepatic release of lipids and is associated with development of insulin resistance in humans with non-alcoholic fatty liver. Fatty liver is a component of the metabolic syndrome of people and is a major risk factor for diabetes development; the occurrence of a similar syndrome in animals is not well documented. However, the proportion of dolphins with fatty liver that also had fasting hyperglycemia (>140 mg/mL) was significantly greater than dolphins without fatty liver (116). Interestingly, dolphins with fasting hyperglycemia and fatty liver were also more likely to have leukocytosis and hyperglobulinemia in the 12-months prior to death than dolphins without fatty liver (116). The presence of the latter findings suggests some dolphins with fatty liver may develop a chronic inflammatory state that resembles steatohepatitis, a severe manifestation of fatty liver syndromes of humans characterized by hepatic inflammation that exacerbates existing insulin resistance. Despite similarities between lipidosis syndromes of dolphins and terrestrial mammals, including cats and humans, insulin resistance has not been specifically demonstrated in dolphins affected with fatty liver. Definitive documentation of insulin resistance and its physiologic and pathologic roles in dolphin health and disease remains aims for future studies.

### Feline obesity

Feline obesity has many parallels with obesity in humans and may serve as a model for the disorder in humans (100). Obesity has been consistently recognized as a strong risk factor associated with feline diabetes in epidemiologic studies, along with other risk factors such as sedentary habits, reproductive status, and assorted environmental factors (123, 124). Yet, obesity in the cat, as in all pet and captive animals, is not a spontaneous disorder. Rather, it is a diet-induced disease that develops as a result of excessive caloric intake (125). The relatively sedentary lifestyle of domesticated or captive carnivores (e.g., pet cat or captive dolphin) has few opportunities to participate in the intense physical activity related to chasing and catching prey, patrolling a territory, and other activities that comprise daily life for free-living individuals. Unlike their wild counterparts, pet and captive carnivores are fed regularly and avoid extended periods of fasting and starvation, which might occur in wild animals subject to cycles in prey availability. Because of its dependence on the availability of excess calories, obesity is expected to occur infrequently, if at all, in free-living populations.

Euglycemic clamp studies in lean and obese cats consistently show insulin sensitivity is decreased in obese cats compared with lean cats (43–45). Body weight was the most significant factor that affects measures of insulin resistance (45). The effect of obesity on insulin resistance is marked and it has been estimated that insulin sensitivity and glucose effectiveness declines by 30% for every 1 kg increase in body weight (45). Along with the hallmark insulin resistance, obese cats develop hormonal changes that parallel those found in obese humans (45, 126). Adiponectin and leptin are hormones produced exclusively by adipose tissue. Adiponectin acts to improve insulin sensitivity (126) and serum concentrations are reduced in obese, insulin resistance humans (127, 128). Serum adiponectin is significantly lower in obese cats compared with lean cats and rise after weight loss (45). Leptin serum concentration is proportional to the mass of adipose tissue in humans (129) and is elevated in obese humans, dogs, and cats (129–131). In obese cats, the serum leptin concentration inversely correlates with insulin sensitivity (114). When obese cats lose weight, leptin concentrations decrease to a level equivalent to the leptin concentration found in the lean cats (45).

## Impact of Domestication and Captivity on Carnivore Carbohydrate Metabolism

In studies with carnivores, it is important to consider possible ways that captivity or domestication might impact “normal” carbohydrate metabolism. Factors such as environment, husbandry, diet, and ability to adapt to captivity are potential confounding factors that must be considered when investigating carbohydrate metabolism using captive populations of bottlenose dolphins. Likewise, the effects of domestication must be taken into account when using the cat as a model for carnivore metabolism. Domestic cats live in a research colony or as pets, consume processed diets, and have a relatively sedentary life style compared with wild felids. Further selective breeding, which has occurred in cats for thousands of years, may have intentionally or unintentionally introduced traits that alter nutrient metabolism. For example, it is possible that the high prevalence of diabetes in pure bred Burmese cats from catteries in Europe and Australia is an unintended outcome of selective breeding since Burmese breed has not been identified as a diabetes risk factor in the U.S. feline population (132, 133). Although the genetic basis for the difference between the two Burmese cat populations is not known, the observation serves to illustrate both the ability of selective breeding to alter metabolic traits over relatively few generations and a possible pitfall when attempting to extrapolate research from a domestic carnivore to the larger group of non-domesticated carnivores (134).

### Impact of captivity/domestication on fasting glucose

Variability in fasting glucose is one example of a possible effect of captivity or confinement on metabolic measurements in carnivores. In three separate studies, fasting glucose in captive bottlenose dolphins was determined to be between 108 and 194 mg/dL (1, 2, 103). The largest study measured serum glucose in 1161 samples from 52 captive bottlenose dolphins and reported mean serum glucose concentrations of 108 and 112 mg/dL in fed and fasted conditions, respectively (2). A second study was a longitudinal observation study that examined 367 samples obtained over a 10+ year period from six dolphins aged ≥30 years, in which mean glucose concentrations were reported to be 121–125 mg/dL, with no significant changes associated with age (103). In the third study, six bottlenose dolphins had fasting mean serum glucose concentrations of 194 and 160 mg/dL determined on separate occasions prior to a feeding challenge (1). Although methodologies used in the studies were slightly different, there is no obvious explanation for the variation observed in the dolphin studies but it is difficult to identify all potential factors that might influence a particular metabolic end point. However, it is intriguing that while no captive population had a mean fasting glucose below 108 mg/dL, a survey of 62 free-living bottlenose dolphins reported a substantially lower mean glucose of 94 mg/dL in the study population (135).

Similarly, the blood glucose of cats may vary unpredictably under some experimental conditions. The propensity for rapid development of hyperglycemia when handled or during interventions is a confounding factor that must be considered when interpreting studies of glucose homeostasis in cats. Investigators have addressed potential “stress hyperglycemia” by using behavioral desensitization techniques to adapt subject cats to the experimental situation prior to obtaining baseline sample (101, 136). Nevertheless, mild to moderate elevation in serum glucose is a well-recognized feline response to stress in clinical or research conditions (101) that may complicate determinations of the “normal” glucose level and glucose tolerance in the cat (101, 137).

### Diet effects on carbohydrate metabolism in domestic/captive carnivores

Diet is another factor that might be expected to influence carbohydrate metabolism in captive and free-living populations. A captive or domestic carnivore may not consume a natural prey-based diet and the substitute diet provided may not replicate exactly the natural diet. The importance of diet for glucose homeostasis is illustrated by the results from a study of captive cheetahs. Cheetahs fed whole prey had different colonic short chain fatty acid profiles and lower colonic propionate concentrations than when they were fed meat alone (138). Colonic propionate can be used by felids as a substrate for gluconeogenesis, as described in a study in the domestic cat (139). There is also evidence that captivity may alter diet choices and feeding ecology of dolphins. Captive dolphins are maintained on a fish-based diet that approximates the free-living diet but may substitute frozen fish/squid for live prey and supplements may be needed to ensure the diet is nutritionally complete. The source of protein (mackerel, herring, squid, etc.), the portion fed, and the frequency of meals may be more uniform in captive diets (140) whereas free-living individuals would encounter prey at irregular intervals, have access to variable amounts of food, and might vary the diet based on prey availability (141).

In cats, the wide variety of available commercial diets formulated using different protein, fat, and carbohydrate compositions, means that diet effects must be carefully controlled for in studies of feline nutrient metabolism. Complete discussion of the complex effect of diet composition on glycemia and metabolism in cats exceeds the scope of this article but the subject has been summarized in detail by others (7). However, pertinent observations concerning possible diet effects on feline carbohydrate metabolism merit a brief discussion. Although cats have no minimum dietary carbohydrate requirement, they are able to effectively digest and absorb diets containing large amounts of processed carbohydrates (104). Digestive signs (diarrhea, gas production, altered motility patterns) indicating dietary intolerance may occur when dietary carbohydrate content exceeds 40% but these signs may reflect osmotic and mechanical effects of large intraluminal sugar concentrations on gut function (18). Consumption of very low carbohydrate diets (7% total carbohydrate content) did not improve glucose tolerance and insulin secretion in non-obese, healthy cats when compared with consumption of diets containing about 25% carbohydrate (142). At the other end of the spectrum, cats fed diets containing 47% carbohydrate demonstrated increased serum glucose (as measured by area-under-the-curve) after a meal but no significant difference in insulin response. Finally is no evidence that dietary carbohydrates cause feline obesity or diabetes (104). In the cat, obesity is more related to total energy consumed than to dietary composition (143, 144).

In the domestic cat, substantial elevations in blood glucose are achieved during challenge tests, such as the intravenous glucose tolerance test, due to the rapid injection of large amounts of glucose, a simple carbohydrate (145–147). However, the dietary carbohydrate content does not strongly impact post-prandial blood glucose in cats, except when the diet is directly supplemented with glucose (21, 105). The relatively blunted rise in glucose after feeding occurs because commercial feline diets, even those with moderate carbohydrate level, contain complex rather than simple carbohydrates (104). Complex carbohydrates have a slower rate of digestion and absorption than simple sugars, like glucose, which are rapidly absorbed from the intestine.

### Acquired disorders of carbohydrate metabolism in domestic/captive carnivores

#### Feline diabetes mellitus

Diabetes mellitus in the domestic cat is a frequently encountered endocrinopathy in veterinary medicine but is probably rare in free-living felids and other carnivores. Overt diabetes mellitus – with hyperglycemia and severe insulin deficiency as prominent features – can be considered a disorder of domestication in that affected cats would die without treatment. Thus, among pet cats and the myriad of other domestic and captive species in which diabetes has been reported, the diabetes prevalence is a reflection of the willingness and ability of veterinarians and pet owners to provide care and treatment (148–154). Similarities in the clinical presentation and treatment of naturally occurring feline diabetes and human type 2 diabetes have already been noted. The pathophysiology underlying the development and maintenance of the diabetic state in the domestic cats has reviewed in detail by others, who have noted the strong parallels between carbohydrate and lipid abnormalities in cats and humans with diabetes (155–158).

#### Dolphin hemochromatosis

Hemochromatosis is well documented in dolphins maintained in captivity but is poorly documented in free-living dolphins. Similarities between hemochromatosis of dolphins and humans has already been discussed but there is evidence that hemochromatosis is a disorder of captivity. A study comparing iron indices reported that serum iron, ferritin, and transferrin saturation levels were lower in free-living dolphins than in captive dolphins, suggesting an association between iron disorders and captivity (120).

## Conclusion: Carnivore Carbohydrate Metabolism as a Model for Human Diseases

Knowledge of the genetic, molecular, and physiologic mechanisms that govern carnivore metabolism is incomplete, yet elucidation of their unique metabolism is essential for understanding the physiology, ecology, and husbandry of carnivore species and their evolutionary adaptation to a specialized ecological niche. Likewise, the potential for carnivores to serve as comparative models for human diabetes has yet to be fully realized. The similarities between normal metabolic processes in carnivores and diabetes pathology in humans suggest carnivores may be useful models for human type 2 diabetes. While no single animal model completely reproduces the human disease, the dolphin and cat models represent a unique opportunity to study important features of human diabetes and offer several advantages as research subjects. First, a major advantage is that diabetes does not have to be induced because the metabolism of interest is present in healthy animals. This avoids a drawback of many traditional animal diabetes models in which the disease must be induced, the animals become clinically ill or require extensive treatment. Second, compared with humans, normal carnivores appear to have mild glucose intolerance and reduced insulin sensitivity, which offers opportunity to study the metabolic pathways that underlie glucose intolerance and insulin resistance, two hallmarks of diabetes pathology in humans. Third, the animals are long lived, which permits study of individual animals over many years, easy to maintain in a controlled environment, are cooperative, and accessible for study.

Carnivores are remarkably adapted to their ecological niche as predators (5, 7, 159–161). Carbohydrate metabolism of healthy carnivores, such as the dolphin and cat, differs from metabolism in healthy omnivores, such as humans, dogs, and rodents. As discussed in this review, carnivore metabolism shares important features with pathological changes observed in pre-diabetic and diabetic humans. It is clear that carnivore metabolism is adaptive, having evolved to facilitate efficient consumption of a prey-based diet that is high in protein and low in carbohydrate content. Remarkably, the metabolic adaptations that sustain glucose homeostasis in carnivores are maladaptive in humans, producing insulin resistance and glucose intolerance and leading to myriad undesirable metabolic consequences (100, 160). While much of carnivore metabolism remains undiscovered, past work has provided a firm base from which to pursue new research into novel metabolic pathways of carnivores and their unique role as models for common metabolic disorders that affect humans.

## Author Contributions

Thomas Schermerhorn is the sole author and conceived, researched, organized, and wrote the manuscript.

## Conflict of Interest Statement

The author declares that the research was conducted in the absence of any commercial or financial relationships that could be construed as a potential conflict of interest.
